# Associations of neuroinflammatory parameters with clinical features in patients with mild cognitive impairment and dementia

**DOI:** 10.1192/j.eurpsy.2022.456

**Published:** 2022-09-01

**Authors:** Y. Zorkina, T. Syunyakov, A. Andriushchenko, O. Abramova, M. Kurmishev, G. Kostyuk, A. Morozova

**Affiliations:** 1 V.P. Serbsky National Medical Research Center for Psychiatry and Narcology, Department Of Basic And Applied Neurobiology, Moscow, Russian Federation; 2 Mental Health Clinic No. 1 named after N. A. Alexeev, Research Center, Moscow, Russian Federation

**Keywords:** MoCA, MCI, MMSE, Dementia

## Abstract

**Introduction:**

Mild cognitive impairment (MCI) represent a state of cognitive function between normal aging and dementia and does not always progress to dementia. Neuroinflammation has a key role in the pathogenesis of neurodegeneration. Determining the associations of neuroinflammatory markers in the blood with clinical disease severity may be useful for early diagnosis of cognitive impairment and prediction of the development of severe dementia.

**Objectives:**

The aim of our study was to compare the serum concentration of a panel of inflammatory markers in patients with MCI and dementia as well as their associations with clinical symptoms.

**Methods:**

Patients were evaluated using Mini-Mental State Examination (MMSE), Clock Drawing Test (CDT), Montreal Cognitive Assessment scales (MoCA), Clinical Dementia rating (CDR) and Hospital Anxiety Depression Scale (HADS). We determined the serum concentration of a panel of inflammatory markers (25 units) cytokines, chemokines, growth factors and several others on Mulliplex and prepared multivariate analysis to investigate associations between clinical features and serum concentration.

**Results:**

Patients with dementia had lower scores on scales than the control and MCI groups. MCI patients were equal to the control group, except for the MMSE scale. EGF, eotaxin-1, GRO-α, IP-10, IL-8, MIP-1β, sCD40L, TNF-α, MDC and MCP-1, VEGF were differ between groups. Multivariate analysis identified some neuroinflammatory parameters associated with the severity of the disease.

**Conclusions:**

We identified some neuroinflammatory parameters associated with dementia and MCI. Many of them have been poor described and data is contradictory. It is necessary to investigate these parameters as potential biomarkers of neurodegeneration in further studies.

**Disclosure:**

No significant relationships.

